# Structural similarities between the metacyclic and bloodstream form variant surface glycoproteins of the African trypanosome

**DOI:** 10.1371/journal.pntd.0011093

**Published:** 2023-02-13

**Authors:** Monica Chandra, Sara Đaković, Konstantina Foti, Johan P. Zeelen, Monique van Straaten, Francisco Aresta-Branco, Eliane Tihon, Nicole Lübbehusen, Thomas Ruppert, Lucy Glover, F. Nina Papavasiliou, C. Erec Stebbins

**Affiliations:** 1 Division of Structural Biology of Infection and Immunity, German Cancer Research Center, Heidelberg, Germany; 2 Division of Immune Diversity, German Cancer Research Center, Heidelberg, Germany; 3 Institut Pasteur, Université Paris Cité, Trypanosome Molecular Biology, Department of Parasites and Insect Vectors, Paris, France; 4 Centre for Molecular Biology at the University of Heidelberg (ZMBH), DKFZ-ZMBH Alliance, Heidelberg, Germany; Universiteit Antwerpen, BELGIUM

## Abstract

During infection of mammalian hosts, African trypanosomes thwart immunity using antigenic variation of the dense Variant Surface Glycoprotein (VSG) coat, accessing a large repertoire of several thousand genes and pseudogenes, and switching to antigenically distinct copies. The parasite is transferred to mammalian hosts by the tsetse fly. In the salivary glands of the fly, the pathogen adopts the metacyclic form and expresses a limited repertoire of VSG genes specific to that developmental stage. It has remained unknown whether the metacyclic VSGs possess distinct properties associated with this particular and discrete phase of the parasite life cycle. We present here three novel metacyclic form VSG N-terminal domain crystal structures (mVSG397, mVSG531, and mVSG1954) and show that they mirror closely in architecture, oligomerization, and surface diversity the known classes of bloodstream form VSGs. These data suggest that the mVSGs are unlikely to be a specialized subclass of VSG proteins, and thus could be poor candidates as the major components of prophylactic vaccines against trypanosomiasis.

## Author summary

The African trypanosome is a single-celled parasite causing African Sleeping Sickness in humans and related diseases in animals. It has a unique protein coat made up mostly of a single protein, called the **V**ariant **S**urface **G**lycoprotein, or **VSG**. The African trypanosome possesses several thousand different VSG genes. As the immune system of an infected host learns to recognize one coat protein and kill the trypanosomes, the parasite switches to a different VSG protein that is not recognized by the immune system. This allows the parasite to keep thriving in the host as this process repeats. This paper presents molecular images of a subset of VSG proteins that are on the surface of the organism when it is transferred to hosts by the bite of the tsetse fly—the first step in infection. We show that these so-called metacyclic VSGs are neither special nor unique compared to those VSGs that take over in the bloodstream during the course of infection. These results clarify the nature of these first VSG coats the host is exposed to and have important implications for trypanosome infection and vaccine development.

## Introduction

*T*. *brucei* is a unicellular pathogen and the cause of African trypanosomiasis in both human and cattle in sub-Saharan Africa [1,2]. Transmitted by the tsetse fly (Glossina sp), *T*. *brucei* is an extracellular pathogen, which swims freely in the bloodstream and is continuously exposed to immune system surveillance [3]. The bloodstream form (BSF) of *T*. *brucei* is covered by approximately ten million copies of the **V**ariant **S**urface **G**lycoprotein (**VSG**) molecule, which comprises over 90% of the surface protein of the pathogen [4,5]. While there are over two thousand VSG-encoding genes and pseudogenes in the trypanosome genome, only one VSG is expressed at a given time from one of numerous bloodstream expression sites (BES) [6]. During the course of infection in the mammalian host, the pathogen undergoes antigenic variation, a phenomenon by which cells expressing one particular VSG are cleared by the humoral arm of the host immune system while a sub-population of trypanosomes switch their coat protein to an antigenically distinct variant and thus can no longer be identified and cleared [7]. The parasites with the “new” coat escape the host immune system and proliferate. This results in waves of parasitemia that characterize trypanosome infection[8].

The VSG protein can be roughly divided into three functional units: the signal peptide for delivery to the cell surface (and which is cleaved during translocation), a large N-terminal domain (NTD) that forms the bulk of the molecule, and the smaller C-terminal domain (CTD) that is tethered to the NTD by a flexible linker that can often be removed by endogenous and exogenous proteases [5,9]. The CTD bears the GPI-anchor, which attaches the VSG to the membrane. The N- and C-terminal domains of the VSGs are divided into different sub-categories based on sequence similarity and the positions of the cysteine residues. Five different classifications of VSG NTDs and six types of the CTD have been suggested amongst other classifications from sequence analysis [10–12]. Despite the wide array of antigenically distinct VSGs, there are only six atomic-resolution structures of the NTD that have been published: from *T*. *brucei brucei* VSG1 (MITat 1.1), VSG2 (MITat 1.2 or VSG221), VSG3 (MITat 1.3 or VSG224), VSG13 (MITat 1.13), ILTat1.24, as well as VSGsur from *T*. *brucei rhodesiense*, with three of these determined in the last four years [13–17]. Structures of the smaller, buried, and more flexible CTDs from VSG2 and IlTat1.24 have been determined separately by NMR [18,19].

The published NTD structures show that the tertiary folds of the VSGs share overall conservation, each resembling a dumbbell-shaped entity with upper and lower globular elements (“lobes”) connected by a three-helix bundle core [9]. These lobes are found as inserted sequences that connect the helices, replacing short linkers seen in more minimal bundle architectures. The conservation of this overall tertiary fold has suggested that the distinct antigenicity of the VSGs is produced primarily by the divergence in amino acid sequence, although the recent structure of VSG3 has broadened the structural “diversity space” of these coat proteins [16]. The presence of a post-translational modification (PTM) on VSG3 (an *O*-glycan at the top-surface of VSG NTD) added another layer of complexity to VSG antigenicity, as the glycan was shown to modulate the host immune response. In addition, the more recent structures of VSG13 and VSGsur [17] have shown that many architectural assumptions need reassessment, as these VSGs show significant deviation from the folds of older structures. Finally, the structure of VSG3 and recent biochemical work [20] have suggested that the NTDs of VSGs of this class can adopt monomeric (lower concentration) and trimeric (higher concentration) oligomeric states, in contrast to the strict dimers of other VSGs.

*T*. *brucei* has a complex life cycle which alternates between the mammalian host and its vector, the tsetse fly. The infectious metacyclic form of *T*. *brucei* inhabits the salivary gland of the tsetse fly and is injected into the mammalian host when the vector takes a bloodmeal. Metacyclic cells differentiate further to the rapidly growing bloodstream form soon after delivery into the host. In contrast to bloodstream VSGs, metacyclic VSGs (mVSGs) are expressed from a dedicated monocistronic metacyclic expression site (MES) that is shorter than the expression sites associated genes (ESAGs) containing bloodstream expression sites (BES)[21,22]. A specific subset of VSGs is expressed in the metacyclic stage, identified as mVSGs 397, 531, 559, 636, 639, 653, 1954, and 3591 (for the Lister 427 strain, [23,24]). These mVSGs represent the first and primary antigenic surface that is presented to the mammalian host immune system, an interaction that initially is focused on Th1 (pro-inflammatory type 1) cytokines, neutrophils and NK cell reactions to pathogen-associated molecular patterns (PAMPs), followed by T-cell independent IgM antibody activity. [25–29].

Because of these distinct stages of development and pathogenesis, it has been hypothesized that the mVSGs could manifest differences with bloodstream form VSGs in some manner related to their use in the initial stage of mammalian infection [30]. These could include structural divergence as well as differences in post-translational modifications (PTMs), such as are found in VSG3 [16]. VSG3 is topologically distinct from all previously characterized VSGs, potentially differing in oligomerization states, and as noted above, harbors *O*-linked hexose chains that are potent immune-modulators. This expansion of the “diversity space” of the VSGs raised the intriguing question: Would the mVSGs show additional structural or chemical diversity that might serve the pathogen in the first stages of entry into the host organism from the tsetse fly?

To begin to address these questions, we have solved the high-resolution crystal structures of mVSG397, mVSG531, and mVSG1954 from *T*. *brucei brucei*, strain Lister 427. Our results lead us to conclude that, as a whole, the mVSGs closely resemble the bloodstream form surface coat proteins both in structure and function.

## Methods

### Cloning and Production of *T*. *brucei* strains

mVSG397, mVSG531 and mVSG1954 from *T*. *b*. *brucei* were cloned into the pUC19 plasmid (BioCat, Germany), and introduced into *T*. *b*. *brucei* strain Lister 427 as described below. The mVSG1954 mutant S321A was generated by site-directed mutagenesis using the QuikChange Lightening kit (Agilent Technologies) according to the manufacturer’s protocol. Transfections were performed into a *T*. *b*. *brucei* cell line expressing VSG2, termed 2T1[31]. First, 5–10 μg of purified plasmids were mixed with 100 μl of 3 × 10^7^ cells in Tb-BSF transfection buffer (90 mM phosphate buffer, pH 7.3, 5 mM KCl, 50 mM HEPES, pH 7.3, 0.15 mM CaCl_2_) and electroporated using a Lonza Nucleofector, program X-001. After 8 h of incubation, hygromycin B was added to a concentration of 25 μg/ml. Single-cell clones were obtained by serial dilutions in 24-well plates in standard culture medium. The surviving clones were confirmed by sequencing. RNA was isolated using the RNeasy Mini Kit (QIAGEN). The isolated RNA was treated with DNase in DNase Turbo Kit (Invitrogen) according to the manufacturer’s protocol. Complementary DNA was synthesized with ProtoScript Reverse Transcriptase (NEB) according to the manufacturer’s protocol. Amplification was performed with a forward primer binding the spliced leader sequence and a reverse primer binding in the VSG 3′-untranslated region (UTR) using Phusion high-fidelity DNA polymerase. PCR products were purified by gel extraction from a 1% agarose gel, followed by the NucleoSpin Gel and PCR clean-up kit (Macherey-Nagel), and sent to Eurofins (Ebersberg) for sequencing using the same primers as for the PCR.

### Purification and Crystallization of mVSG397, mVSG531, and mVSG1954

*T*. *b*. *brucei* strains expressing mVSG397, mVSG531 and mVSG1954 were cultivated *in vitro* in HMI-9 medium (formulated as described [17]), supplemented with 10% fetal calf serum (Gibco), L-cysteine and β-mercaptoethanol. Cells were cultured at 37°C and 5% CO_2_. VSGs were purified according to established protocols [32]. Briefly, cells were pelleted and then lysed in 0.2 mM ZnCl_2_. The lysis mixture was centrifuged and the pellet containing the membrane material was resuspended in prewarmed (42°C) 20 mM HEPES pH 7.5, 150 mM NaCl. Following a second centrifugation, supernatant containing VSG protein was loaded onto an anion-exchange column (Q-Sepharose Fast-Flow, GE Healthcare), which had been equilibrated with 20 mM HEPES pH 7.5, 150 mM NaCl. The flow-through and washes fractions containing highly pure VSG was concentrated in an Amicon Stirred Cell with 10 kDa MWCO membrane. The concentrated VSGs were subjected to size exclusion chromatography as the last clean-up step using HiLoad 16/600 Superdex 200 column (GE Healthcare) in AKTA pure chromatography system (GE Healthcare).

Concentrated mVSG397 was digested with trypsin in a 1:100 trypsin:VSG ratio on ice for 1 hour. The mVSG397 NTD was purified using a gel filtration chromatography Superdex 200 Increase 10/300GL column (GE Healthcare) equilibrated with 10 mM Hepes-NaOH, 150 mM NaCl. The fractions containing the protein were concentrated to 5 mg/ml. Crystals were grown by vapour diffusion using 100 mM SPG buffer (pH 5.2) and 21% (W/V) PEG 1500 and soaked in 100 mM SPG buffer (pH 5.2), 21% (W/V) PEG 1500 and 10% (W/V) PEG 400 before flash cooling to 100 K (−173.15 °C). For phasing, crystals were grown by vapour diffusion using 100 mM SPG buffer (pH 5.2) 24% (W/V) PEG 2000 MME and shortly soaked in 100 mM SPG buffer (pH 5.2), 24% (W/V) PEG 200 MME, 50 mM KI and 25% (W/V) ethylene glycol before flash cooling to 100 K (−173.15 °C). Data sets were collected at the Paul Scherrer Institut, Swiss Light Source, Villingen, at a wavelength of 1.0 Å or 2.066 Å (native and KI soaked crystals, respectively). The structure was solved using single wavelength anomalous diffraction (SAD) with the software CCP4I2-CRANK2 [33], and an initial model was built with Buccaneer[34]. This model was used for molecular replacement in PHENIX [35,36] against the higher resolution native data. The structure was refined and built using PHENIX, COOT [37] and PDB-REDO [38].

Native crystals of mVSG531 were grown by vapor diffusion using hanging drops formed from mixing a 1:1 volume ratio of the full-length protein at 7 mg/ml with an equilibration buffer consisting of 25% PEG 1500, 0.1M MMT buffer (DL-Malic acid, MES monohydrate, Tris) pH 6.5, 3% glucose. Crystals of only the NTD appeared after 3 weeks at room temperature. For cryoprotection, crystals were transferred directly into a buffer with 10% glycerol and flash-cooled immediately afterward to 100 K (−173.15 °C). Crystals formed in the space group P2_1_ with four dimers of VSG531 in the asymmetric unit (total of eight independent copies of VSG531 NTD). Data were collected at the European Synchrotron Radiation Facility (ESRF) in Grenoble at beamline ID29 and processed onsite through the EDNA framework Fast Processing System (PMID: 19844027). The structure was solved by molecular replacement using the model of VSG1 (MITat1.1, PDB ID 5LY9) with PHASER as implemented in the PHENIX package [39]. Iterative cycles of model building with the PHENIX [40], manual adjustment in COOT, and refinement in PHENIX let to a final model with and R/Rfree of 21.34%/24.38% with no Ramachandran outliers. An N-linked carbohydrate is present in each monomer at residue N295.

The concentrated mVSG1954 was subjected to limited proteolysis using trypsin in 1:50 trypsin to VSG ratio at 4°C for 3 hours to remove the more flexible C-terminal domain. The proteolysis reaction was stopped by adding phenylmethylsulfonylfluoride (PMSF) to 1 mM final concentration. The N- and C-terminal domains were separated by size exclusion chromatography using HiLoad 16/600 Superdex 200 column (GE Healthcare) in AKTA pure chromatography system (GE Healthcare). Native crystals were grown by vapor diffusion using sitting drops formed from mixing a 1:1 volume ratio of the protein at 10mg/ml with an equilibration buffer consisting of 20% PEG 3350, 0.4M NaCl at the University of Heidelberg Crystallization Platform. Crystals appeared after 19 hours at 18°C. The native crystals diffracted to 2.28 Å at Paul Scherrer Institut, Swiss Light Source, Villigen, Switzerland. The derivative crystals were grown by vapor diffusion using sitting drops formed from mixing a 1:1 volume ratio of the protein at 10mg/ml with an equilibration buffer consisting of 25% PEG 1500, 0.4M NaBr for halide phasing. The crystals appeared after 3 days following incubation at 22°C. For cryoprotection, crystals were transferred directly into a stabilization buffer with 0.75M NaBr and 20% glycerol and flash-cooled immediately afterward to 100 K (−173.15 °C). Anomalous X-ray diffraction data were collected at 0.9198 Å wavelength to obtain single-wavelength anomalous dispersion (SAD) dataset. The best diffracting derivative crystal diffracted to 1.68 Å. Crystals formed in the space group P321 with a monomer of mVSG1954 in the asymmetric unit. The substructure atoms were found using SHELX [41] in the CCP4i2 [42] crystallography software suite. Initial model building was performed in Arp/wArp [43] and BUCCANEER in the same suite. Subsequently, the model was further refined in PHENIX. However, after data quality analysis, the last 300 frames in the dataset were opted out from the processing in xia2/DIALS [44–46] to avoid incorporating data with radiation damage. The refined structure was used to perform molecular replacement with PHASER in PHENIX on the new reprocessed data. Subsequent refinement was performed in PHENIX. Cycles of manual model examination using COOT and improvement with automated refinement completed the model. Final model with and R/Rfree of 20.9%/21.3% with no Ramachandran outliers. An N-linked carbohydrate is present in each monomer at residue N374.

### Suramin resistance assays

Performed as previously described [17].

### Mass Spectrometry with Purified mVSG1954 Wild Type and S321A

Protein samples were loaded on a self-packed reversed-phase column (0.8 × 2 mm, Poros R1) for desalting and concentration using 0.3% formic acid (0.3 ml/min). After 3 minutes proteins were eluted with 40% isopropanol, 5% acetonitrile, 0.3% formic acid (0.04ml/min) and analyzed with a QTof mass spectrometer (maXis, Bruker Daltonics) after electrospray ionization. Data were deconvoluted using the ESI Compass 1.3 Maximum Entropy Deconvolution Option (Bruker) to determine the molecular weight of the proteins.

### Protein thermal stability measurements

The thermal stability of the wild type mVSG1954 and the S321A mutant (both full length VSGs) were determined using the Prometheus NT.48 nanoDSF (NanoTemper Technologies). The proteins at 2 mg/ml were loaded into standard capillaries. The intrinsic protein fluorescence was measured at 330 nm and 350 nm with a temperature gradient ranging from 20 to 90°C at a rate of 1°C/min. The data were analysed with the PR.ThermControl software provided with the instrument.

### Circular dichroism spectroscopy

The circular dichroism spectra of the wild type mVSG1954 and the S321A mutant (both full length VSGs) were recorded between 260 nm and 190 nm at 20°C at a concentration of 0.5, 1, and 2 mg/ml in 20 mM HEPES pH 8.0 and 150 mM NaCl. The spectra were recorded using a Jasco J-815 CD spectrometer with a 50 nm/min scan speed, digital integration time of 1 sec with a 1 nm bandwidth and 10 accumulations.

## Results

### Structure-focused classification of Metacyclic and Bloodstream VSGs

**Fig 1:**
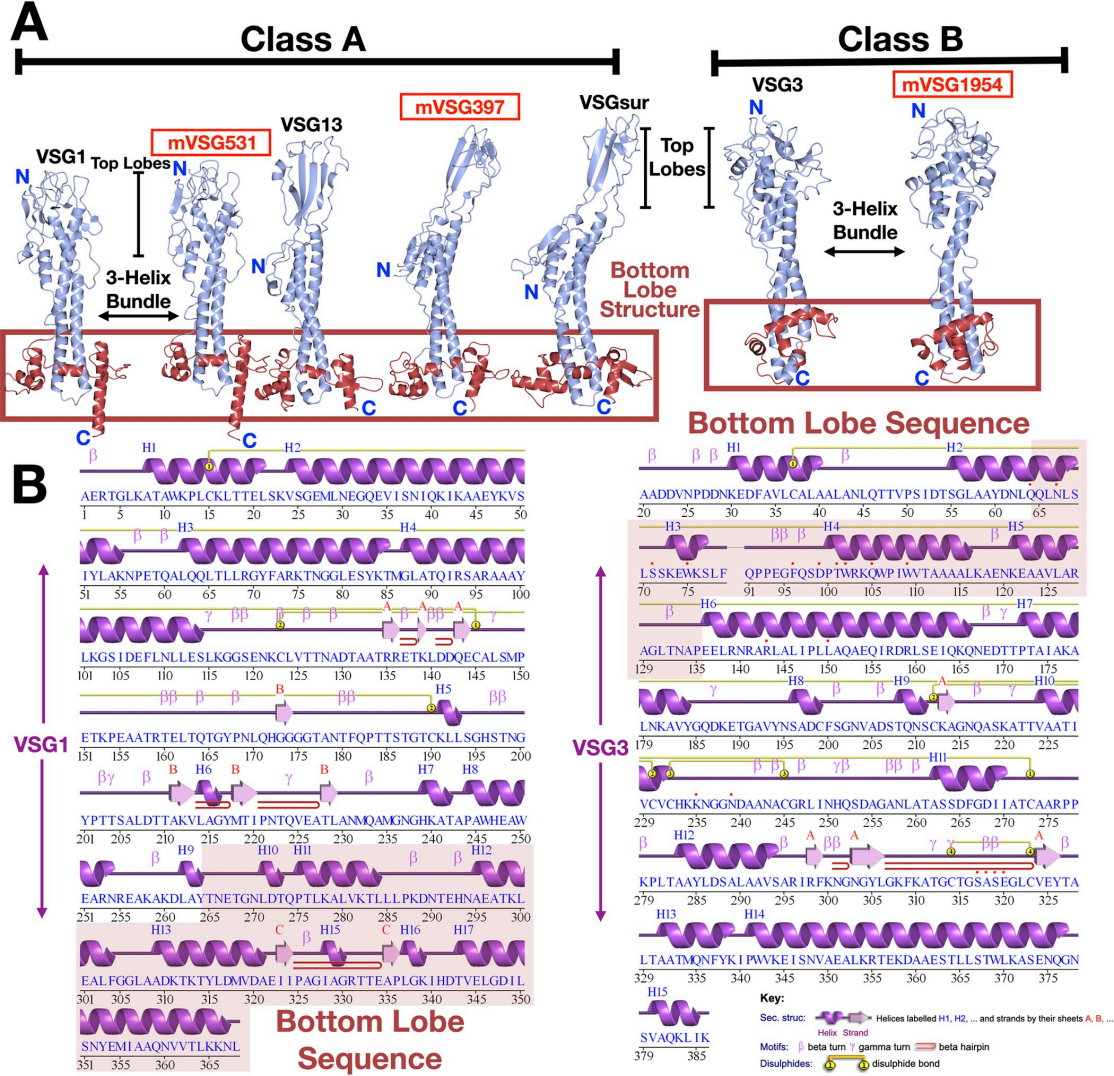
Structural classification of VSG proteins. (A) Two broad superfamily classes of VSGs identified through sequence analysis and defined by structural topology are shown here with representative structures. VSG monomers are shown as ribbon diagrams colored in light blue. Structures reported in this manuscript are denoted in red. The lower, bottom lobe subdomains of the VSGs are outlined with a red box. Structures were drawn with CCP4*mg* [57]. (B) Sequence and structurally-determined secondary structure of two representative VSGs from Class A (VSG1) and Class B (VSG2). The sequence region that forms the bottom lobe is indicated by red highlighting. Secondary structure was illustrated with PDBSUM [58].

Using structural topology as a guide, the known VSG NTD structures can be divided into two “super-families” that correlate well with previous divisions based on primary sequence analyses (Classes A and B), while also providing an architectural explanation for the class division. Class A includes the largest set of members that have been structurally characterized: VSG1, VSG2, ILTat1.24, VSG13, and VSGsur (Fig 1A and 1B). This class possesses bottom lobes of globular secondary structure (near the membrane proximal portion of the 3-helix bundle of the VSG NTD; Fig 1A) that are composed of primary sequence that is located close to the C-terminal portion of the NTD. In contrast, Class B is topologically distinct, possessing a bottom lobe that is comprised of sequence from the N-terminal portion of the molecule (Fig 1). This suggests that while there can be many subdivisions within each of these clans, there is a broad overarching bifurcation of the VSGs by protein structural topology that is dictated by the position of the amino acid sequence encoding the bottom lobe structure.

As a subclass, the metacyclic VSGs studied here do not deviate from this pattern. VSGs 397 and 531 fall within Class A by homology analysis, whereas VSG1954 is a member of Class B. More specifically, by using the classification system outlined in Cross et al. [10], mVSG397 belongs to Class A1 (similar to VSG522, which shares 72% sequence identity with *Trypanosoma brucei rhodesiense* VSGsur (GenBank: ATI14856), whose unusual structure was recently determined [17]), mVSG531 to Class A2 (the same as VSG1 and VSG2), and VSG1954 to Class B.

### Crystal structure of mVSG397 shows close homology to *T*.*b rhodesiense* BSF VSGsur

**Fig 2:**
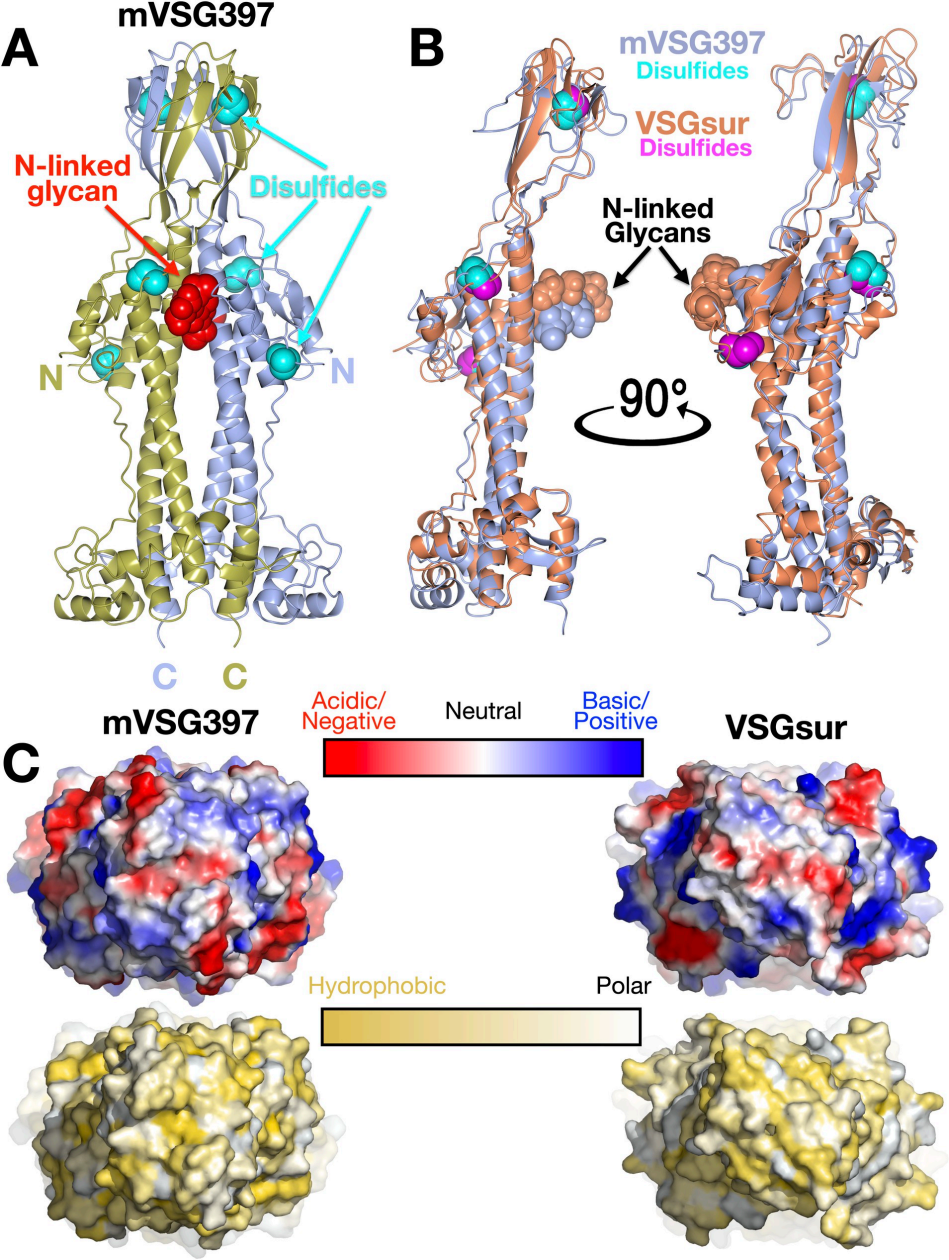
Crystal structure of mVSG397. (A) Ribbon diagram of the (crystallographic) dimer of mVSG397, the individual monomers colored gold and light blue. The N-linked glycans are displayed as red space-filling atoms and disulfide bonds are shown in cyan. ‘N’ and ‘C’ indicate the N and C termini of each monomer. Structures drawn with CCP4*mg* [57] (B) Structural alignment of monomers of VSGsur (orange) and mVSG397 (light blue) with corresponding glycans and disulfides shown in space filling representation, the sugars colored the same as the protein to which they are linked, the disulfides in cyan (mVSG397) and magenta (VSGsur). Structures drawn with CCP4*mg* [57] (C) 90-degree rotation of the structures in (A) and (B) to view the “top” of the VSGs, rendered as molecular surfaces. Top row shows the surface colored by electrostatic potential (red indicating acidic/negatively charged, blue indicating basic/positively charged, and white neutral, all surfaces scaled identically). The bottom row is colored by the Eisenberg hydrophobicity scale [59], where yellow indicates hydrophobic and white polar. Molecular surfaces are generated by PyMOL [60].

To extend the comparison between the mVSGs and the BSF VSGs beyond sequence analysis, we determined the crystal structure of Class A1 mVSG397 to 1.26Å resolution (Methods, Figs 2 and S1A and S1 Table). As noted, sequence clustering analysis classified this VSG similarly to VSGsur, and the structure confirms the architectural similarity. Like VSGsur, mVSG397 possesses several distinct conformational features strongly distinguishing it from known structures of both Class A2 and Class B VSGs, while retaining the bottom lobe topological relationship with those Class A structures. A prominent distinction is the top lobe of the VSG, which consists of a large, globular subdomain—a tightly twisted β-sheet in the monomer that forms a β-sandwich in the dimer (Fig 2A and 2B). Secondly, unlike the VSG1, VSG2, and VSG3 which possess N-linked glycans at the bottom lobes, the sugar in mVSG397 is positioned similarly to VSGsur, located directly below the top lobe, nearly two-thirds the distance from the bottom of the NTD. A third similarity to VSGsur is the disulfide bond distribution throughout the length of the NTD, whereas the other known VSGs contain such bonds clustered in the upper portion of the top lobe. Finally, as with VSGsur, the N-terminus of the protein is not at the uppermost surface, but located below the top-lobe and N-linked glycan, possibly buried significantly when packed on the membrane. Overall, mVSG397 and VSGsur align with a root mean square deviation (RMSD) of 2.33 Å over 334 residues (Fig 2B). Consistent with the functional VSGs possessing antigenic distinctiveness to the immune system, the molecular surface of mVSG397 differs appreciably from that of VSGsur in topography, electrostatic and hydrophobic properties (Fig 2C).

The structure of VSGsur was published together with the structure of VSG13, the latter a VSG also possessing a large β-sandwich top lobe, an N-linked glycan directly underneath this subdomain, and disulfides distributed over the length of the NTD. However, the β-sheet domains of VSGsur and VSG13 do not align well (that of VSG13 being less twisted and much broader), and mVSG397 resembles VSGsur in this region and not VSG13. This is consistent with the sequence clustering classification of these A-type VSGs into different subgroups, A1 for both VSGsur and mVSG397 but A3 for VSG13 [10].

Finally, it should be noted that variant VSGsur from *T*. *b*. *rhodesiense* binds to the trypanolytic drug suramin, thereby conferring heighted resistance to the drug [17]. While mVSG397 possesses less than 30% sequence identity with VSGsur in the NTD, the question arises of whether it could bind suramin as well. Suramin binds VSGsur in a large cavity formed between the helices of the three-helix bundle, directly below the N-linked glycan ([17], S2A Fig). However, no such large cavity exists in mVSG397 (S2B Fig). Indeed, trypanosome strains expressing VSG397 are not resistant to suramin like VSGsur (S2C Fig). A better candidate for suramin binding in the Lister 427 strain would be VSG522, which possesses 72% sequence identity with VSGsur, including key binding residues to the drug. An AlphaFold [47] model of the VSG522 dimer aligns with a RMSD of 1.2Å over 719 residues with the VSGsur dimer (S2D Fig). Importantly, the critical suramin binding residues of VSGsur (H122 from each monomer) have counterparts in VSG522 (H93) that are in the same location on the helical bundle in the cavity (S2D Fig).

### Crystal structure of VSG531 shows close homology to class A2 members

**Fig 3:**
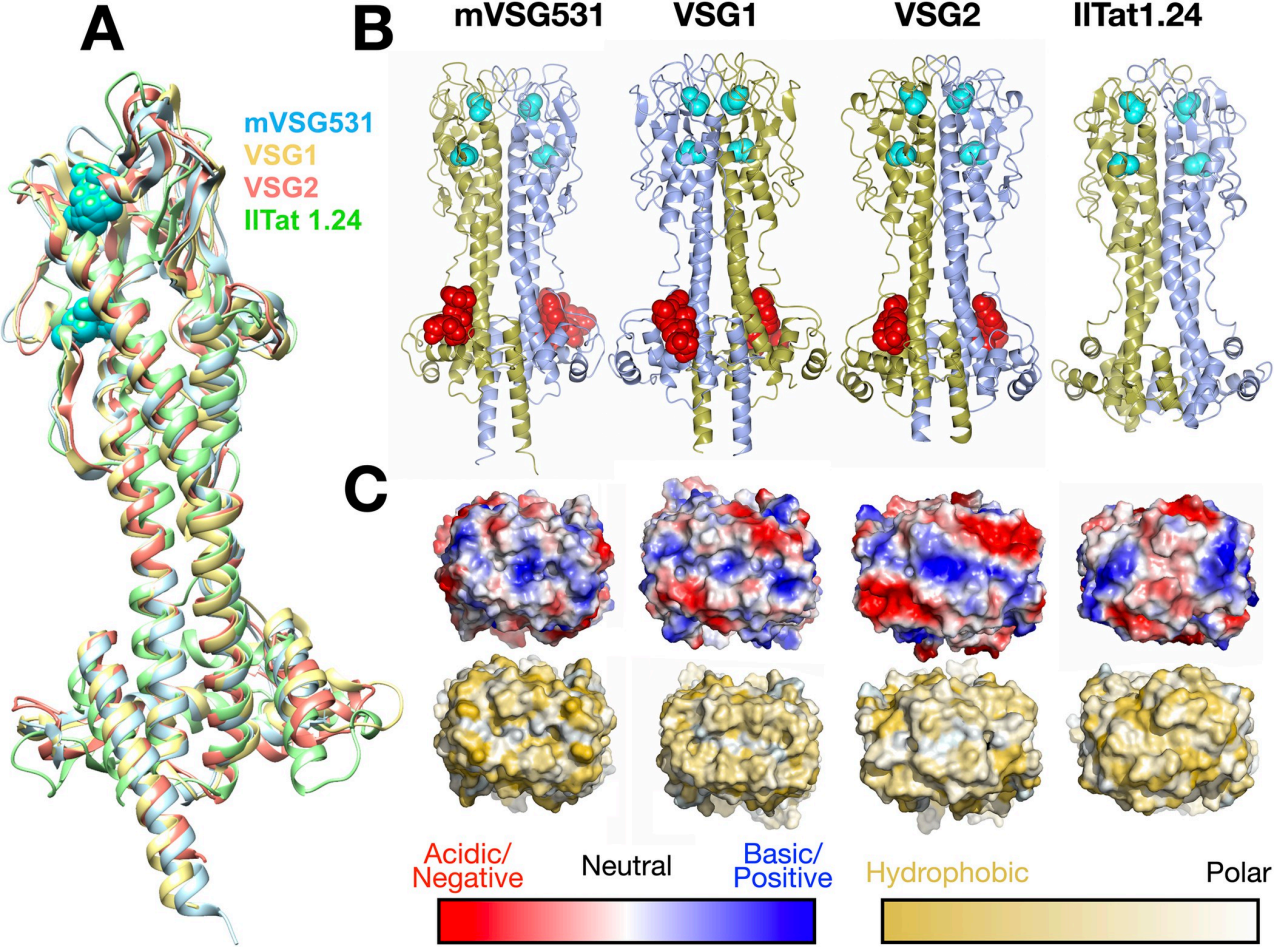
Crystal structure of mVSG531. (A) Superposition of class A2/N2 monomers of mVSG531, VSG1, VSG2, and IlTat1.24 produced using DeepAlign in the RaptorX structure alignment server [48,49]. Images of protein structures were generated and edited using CHIMERA [61]. (B) Side-by-side comparison of different class A VSG dimer highlighting common fold, disulfide placement (cyan spheres), and N-linked glycan (red spheres). Structures drawn with CCP4*mg* [57]. (C) 90-degree rotation of the structures in (B) to view the “top” surface of the VSG, rendered as a molecular surface. Top row shows the surface colored by contact potential (red indicating acidic/negatively charged, blue indicating basic/positively charged, and white neutral, all surfaces scaled identically). The bottom row is colored by the Eisenberg hydrophobicity scale [59], where yellow indicates hydrophobic and white polar. Molecular surfaces are illustrated with PyMOL [60].

We also crystallized mVSG531, predicted to be of the Class A2 group, determining its structure to 1.95Å resolution (Figs 3 and S1B and S1 Table and Methods). Consistent with bioinformatic analyses, mVSG531 is very similar to the structures of other Class A2 members solved from BSF VSGs, particularly VSG1 and VSG2, aligning to each with a RMSD of 2.1Å over 354 residues and 2.4Å over 347 residues, respectively (Fig 3A, [48,49]). In fact, with 35% sequence identity, the structure of VSG1 is similar enough to mVSG531 that we were able to use it to phase the diffraction data through molecular replacement (see Methods, S3 Fig). Most of the divergence between VSG531 and VSG1/VSG2 occurs in the top lobes, with the bottom lobe showing higher conservation, and the three-helix bundle the most conserved region of the molecule (RMSD close to 1Å, S4 Fig).

Similarities to VSG1 and VSG2 extend beyond the overall conservation in the fold. The positions of the two cysteine disulfides bonds in the top lobe of the N-terminal domain closely overlap with several of those in structures of bloodstream Class A2 members (Fig 3A and 3B), although this does not hold for all Class A members, such as A3 member VSG13 and, as discussed above, Class A1 mVSG397 and VSGsur. The N-linked sugars of VSG1, VSG2, and mVSG531 are located in very similar positions in the lower lobe as well (Fig 3B). Finally, the molecular surfaces of different VSGs show high variance in shape, charge distribution, and polar/hydrophobic character (Figs 2C and 3C), regardless of which subgroup to which they belong.

### Crystal structure of mVSG1954 shows close homology to class B members

To further compare mVSGs and BSF VSGs, we characterized the structure of Class B member mVSG1954 at 1.68 Å resolution (Methods, Figs S1C and 4). mVSG1954 and VSG3 (also class B) show a good degree of structural conservation, aligning with an RMSD of 4.6Å over 308 residues). The conservation extends to the location of the four cysteine disulfides bridges concentrated at the top-lobe of the monomer.

**Fig 4:**
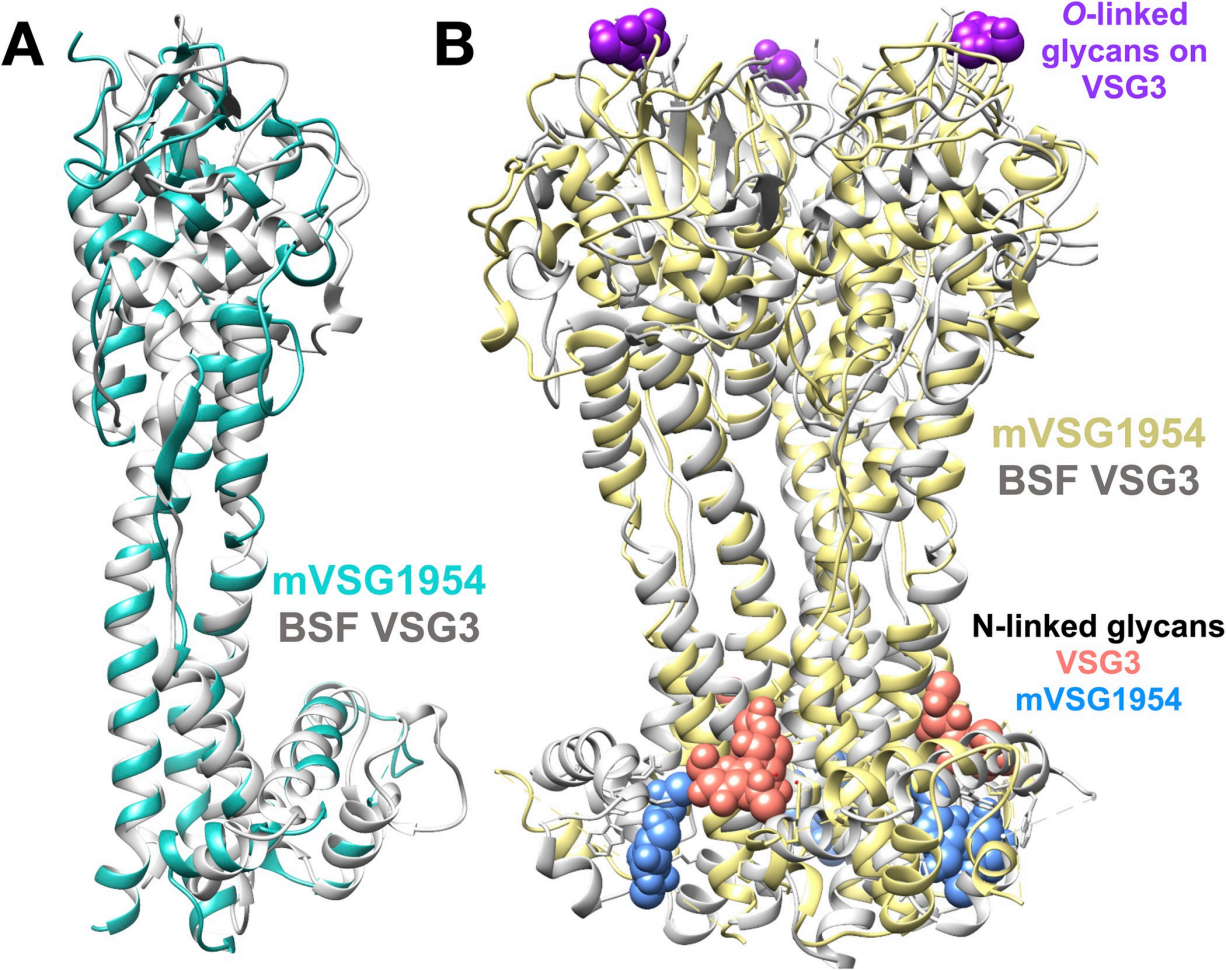
Crystal structure of mVSG1954. (A) Superposition of class B mVSG1954 (aquamarine) and BSF VSG3 (grey) monomers by DeepAlign in RaptorX structure alignment server. (B) Structural superposition of the crystallographic trimers of mVSG1954 (yellow) and VSG3 (grey) performed by SSM Superpose function in COOT [37,62]. The *O*-linked sugar on the top lobe of VSG3 is shown in purple as a space-filling atomic representation, whereas the N-linked glycans are shown in salmon (VSG3) and blue (mVSG1954), respectively. Images of protein structures were generated and edited using CHIMERA [61].

As with VSG3, and in contrast to Class A VSGs, mVSG1954 behaves as a monomer in solution (S1C Fig) and crystallized as a monomer in the asymmetric unit of the crystal. The ability of mVSG1954 to exist as a monomer is further corroborated by SEC-MALS analysis (size exclusion chromatography coupled to multi-angle light scatting) [20]. Also, like VSG3, while a monomer in the asymmetric unit, the mVSG1954 NTD exists as a crystallographic trimer that is highly similar to that of VSG3 (Fig 4B and S1 Table). Of note is that SEC-MALS data of Class B VSG9 indicates that it forms a trimer in solution [20]. Altogether, these data argue that, diverging from the dimers of Class A VSGs, members of Class B may adopt a trimeric oligomeric state, at least in solution and crystals, and possibly on the membrane.

In contrast to the structure of VSG3, the NTD of mVSG1954 does not show any evidence in the crystals of *O*-linked glycosylation, despite possessing a close consensus sequence to the motif shown previously to often contain this PTM [16]. Using intact protein mass spectrometry, the absence of such a sugar on the conserved loop at S321 was confirmed (the residue predicted to be glycosylated from the consensus sequence), although a hexose residue was found on the full length protein, even after PNGase treatment (S5 and S6 Figs). This additional hexose may be located on the highly glycosylated CTD or on a novel site in the NTD that was, nonetheless, not seen to be glycosylated in the crystals. Furthermore, because VSG3 sugars have been shown to be heterogeneous [16] and labile [50], it cannot be ruled out that carbohydrate modifications were lost during the process of purification, crystallization, and/or preparation for mass spectrometry.

## Discussion

The evolution of the host antibody response and parasite immune evasion mechanisms is a continuous arms race. *T*. *brucei* overcomes host immunity during the course of infection through a repetitive process of switching VSGs to antigenically distinct versions. The large archive of VSG genes available in *T*. *brucei’s* genome thereby enables extended survival in the host by continuous antigenic variation [51,52]. Aside from amino acid sequence diversity, recent literature has shown that VSGs are also diverse in their tertiary structure organization, oligomerization states, and post-translational modification, factors that can modulate the immune response.

This study focuses on the metacyclic life stage of *T*. *brucei* in which it inhabits the tsetse fly’s salivary gland and is subsequently transmitted into the mammalian host when the fly takes a blood meal. Like BSF cells, MCF cells also express VSGs on their surface [53]. When injected into the mammalian host, the mVSGs expressed on the parasites’ surfaces are retained up to seven days before differentiating into BSF cells that express VSGs from telomeric BESs [54,55]. In the Lister 427 *T*. *brucei* lab model strain used broadly in the study of the African trypanosome, there have been eight VSGs identified that reside in MESs and which are therefore expressed in MCF cells, but structural and functional information on mVSGs has been lacking, particularly whether this unique life cycle stage presents a coat with intrinsic differences from those in the BSF.

This work begins to address these questions at the structural level. The three structures presented here cover a broad range of VSG types, including two divergent Class A molecules and one Class B. Although the metacyclic stage presents unique challenges to the pathogen, these results indicate that mVSGs nonetheless adopt similar three-dimensional properties as their bloodstream counterparts. These data suggest that the mVSGs are likely not a specialized subclass of VSG proteins, but instead are derived from the vast genomic archive available through various gene recombination events. This is supported by the fact that the identity of the mVSGs, and even their number, varies dramatically between trypanosome strains. Barring the occurrence of specialized PTMs or other alterations not detected in the BES-produced proteins in this study, the VSG component of the metacyclic coat is not radically different than the BSF.

As MCF cells are the first antigenic surface presented to the mammalian host, and as their mVSG repertoire is limited (eight mVSGs in Lister 427), mVSGs might appear to be an attractive target in the context of prophylaxis treatment (e.g. a vaccine). However, the findings herein that an mVSG itself appears to be “just another” VSG structurally, together with previous studies that have identified mVSG-like proteins expressed within BESs in populations of VSG switchers [56], suggests that the repertoire of mVSGs (or rather, of MES-resident VSGs expressed within MCFs) in nature can be (or can become) just as diverse as that of BES-expressed variants. It is therefore unlikely that mVSGs vaccines can be an effective prophylactic proposition in the context of trypanosomiasis.

## Supporting information

S1 Fig Purification, Crystallization, and Representative Electron Density of mVSGs. Summary of various steps in the crystallographic structural solution. Panels showing the gel filtration chromatogram (Superdex 200, Methods) of purified (A) mVSG397, (B) mVSG531, and (C) mVSG1954 with a coomassie stained SDS-PAGE gel of the final material used for crystallization. Images of crystals grown in hanging drops, X-ray diffraction, and the final model 2Fo-Fc electron density contoured at 1σ are added alongside the chromatograph. (D) 2Fo-Fc electron density contoured at 1σ of the N-linked glycans observed. Electron density illustrated with COOT.
(TIF)

S2 Fig Structural and Functional Comparisons of mVSG397 with VSGsur. (A) and (B) Ribbon diagrams drawn with CCP4Mg showing that the pocket between monomers is much smaller in mVSG397 as compared to VSGsur. Note that the dimer of VSG397, like that of VSGsur shown, is crystallographic, as VSG397 crystallizes as a monomer in the asymmetric unit (like unbound VSGsur, PDB ID 6Z7A). However, a standard class A dimer is present in the crystals with the dimeric axis aligned with a crystallographic two-fold axis of symmetry (again, as seen with unbound VSGsur). Such an arrangement, monomer in the asymmetric unit, alignment of the dimer axis with a crystallographic two-fold axis of rotation, has also been seen with VSG2. (C) Suramin resistance assays showing that mVSG397 does not confer resistance to suramin. Differences between VSG397 and VSG2 are not statistically significant (two-tailed P = 0.3029) whereas the difference between VSG397 and VSGsur are statistically significant (two-tailed P<0.0001). (D) AlphaFold was used to predict the structure of a VSG522 dimer with ColabFold. The panel shows an alignment of VSGsur (gold) and VSG522 (blue) with the key suramin-binding residues of VSGsur (H122 from each monomer) and the corresponding H93 of VSG522 colored as the main chains.
(TIF)

S3 Fig Atomic resolution structure of mVSG531. Molecular replacement was performed by PHASER-MR on the native crystal dataset using BSF VSG1 as search model to determine the structure of mVSG531 (Methods). (A) Eight molecules of mVSG531 was found in the crystal asymmetric unit. (B) N-linked glycan is attached at Asn295 at the dimer’s bottom lobe (salmon pink spheres). Two disulfide bonds are observed at the top lobe of each molecule (green spheres). Images of protein structures were generated and edited using CHIMERA.
(TIF)

S4 Fig Structure alignment between mVSG531, VSG2, and VSG1 reveals structure similarity between mVSG531 and bloodstream VSGs in Class A. (A) Overall structural alignment of mVSG531, VSG2 (PDB code: 1VSG), and VSG1 (PDB code: 5LY9) monomers (yellow: VSG2, blue: VSG1, salmon pink: mVSG531, green: disulfide bonds) (B) Three-helix bundle core (left) and the bottom lobe (right) structural alignment. Structural alignment was performed by DeepAlign in RaptorX structure alignment server. (r.m.s.d.: root mean square deviation uGDT/GDT: (unnormalized) global distance test, TM-score: template modeling score). Images of protein structures were generated using CHIMERA.
(TIF)

S5 Fig mVSG1954 by mass spectrometry. Intact mass spectrometry was performed on full length mVSG1954 wildtype and S321A (A and C). Both protein constructs are also treated with PNGaseF to cleave the N-linked glycans (B and D). Mass differences of 203 Da and 162 Da was observed in the full-length mVSG1954 wildtype (A), which correspond to N-acetylglucosamine and hexose molecules, respectively. When the sample was treated with PNGaseF (B), only the 162 Da mass difference was observed, indicating the loss of N-linked glycan and the possible presence of O-linked glycan. This was examined by mutating the putative O-glycosylation site at S321 to alanine, which cannot be O-glycosylated. A 16 Da mass difference was observed between the full length mVSG1954 wildtype (A) and S321A (C), indicating the serine to alanine mutation. However, when treated with PNGaseF (D), the 162 Da mass difference still presents, indicating that the protein is not O-glycosylated on that residue. Since the material is FL VSG1954, it is possible the loss of a mass equal to a hexose could be from the CTD GPI anchor or from another site on the NTD that was, nonetheless, not seen in the crystals.
(TIF)

S6 Fig Examination of VSG1954 mutant: thermal unfolding and circular dichroism. (A) thermal stability curves of the wild type (WT) mVSG1954 and the VSGS321A mutant (Methods). (B) Circular dichroism spectra of the WT and mutant VSG1954 (Methods).
(TIF)

S1 Table Crystallographic Statistics. (DOCX)
