# Supplementary figures and images for: Structural similarities between the metacyclic and bloodstream form variant surface glycoproteins of the African trypanosome
Source: PLoS Negl Trop Dis. 2023 Feb 13;17(2):e0011093. doi: 10.1371/journal.pntd.0011093 (PMC9956791; doi:10.1371/journal.pntd.0011093)

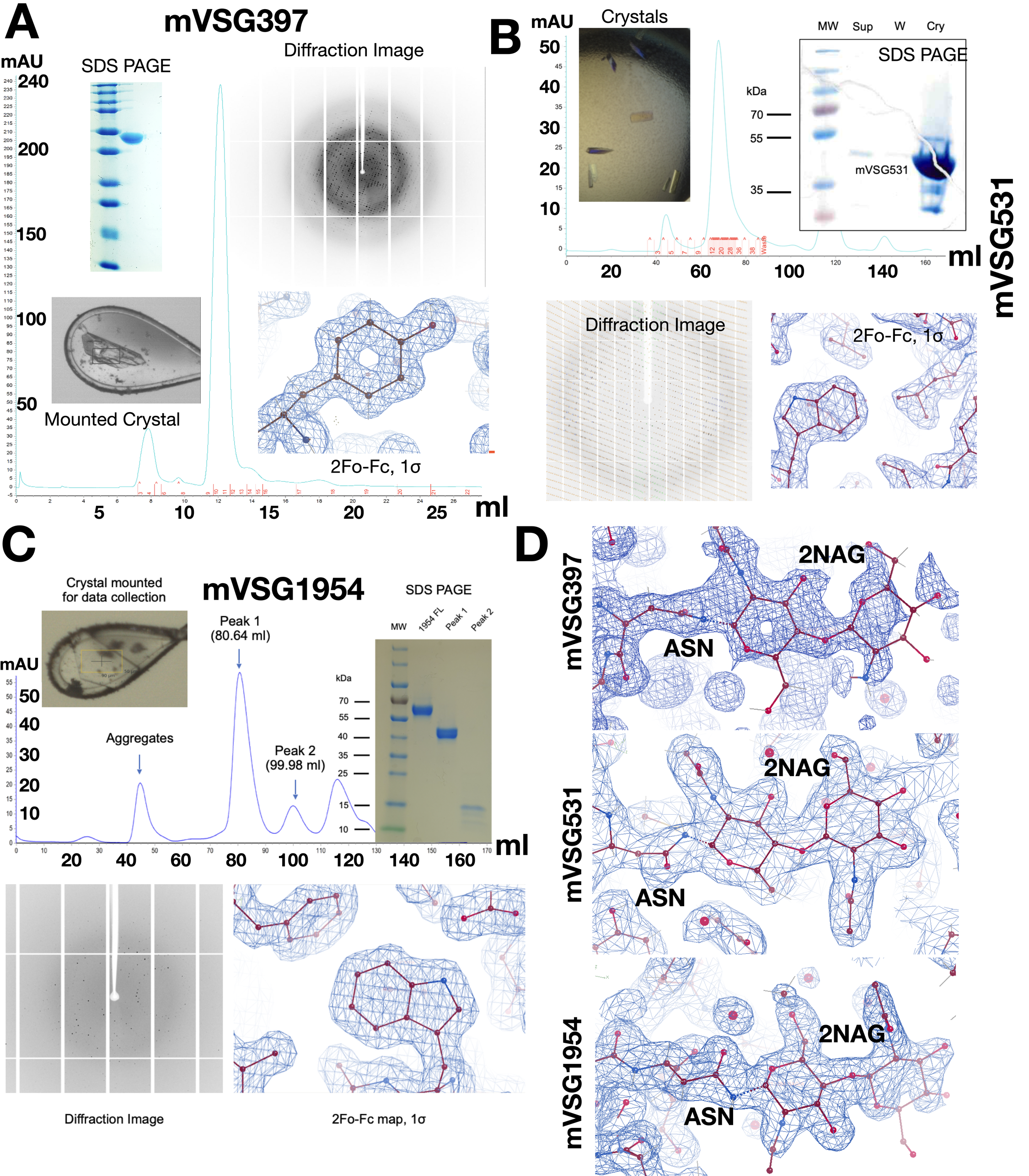

Supplement: S1 Fig — Summary of various steps in the crystallographic structural solution. Panels showing the gel filtration chromatogram (Superdex 200, Methods) of purified (A) mVSG397, (B) mVSG531, and (C) mVSG1954 with a coomassie stained SDS-PAGE gel of the final material used for crystallization. Images of crystals grown in hanging drops, X-ray diffraction, and the final model 2Fo-Fc electron density contoured at 1σ are added alongside the chromatograph. (D) 2Fo-Fc electron density contoured at 1σ of the N-linked glycans observed. Electron density illustrated with COOT. (TIF) [file pntd.0011093.s001.tif]

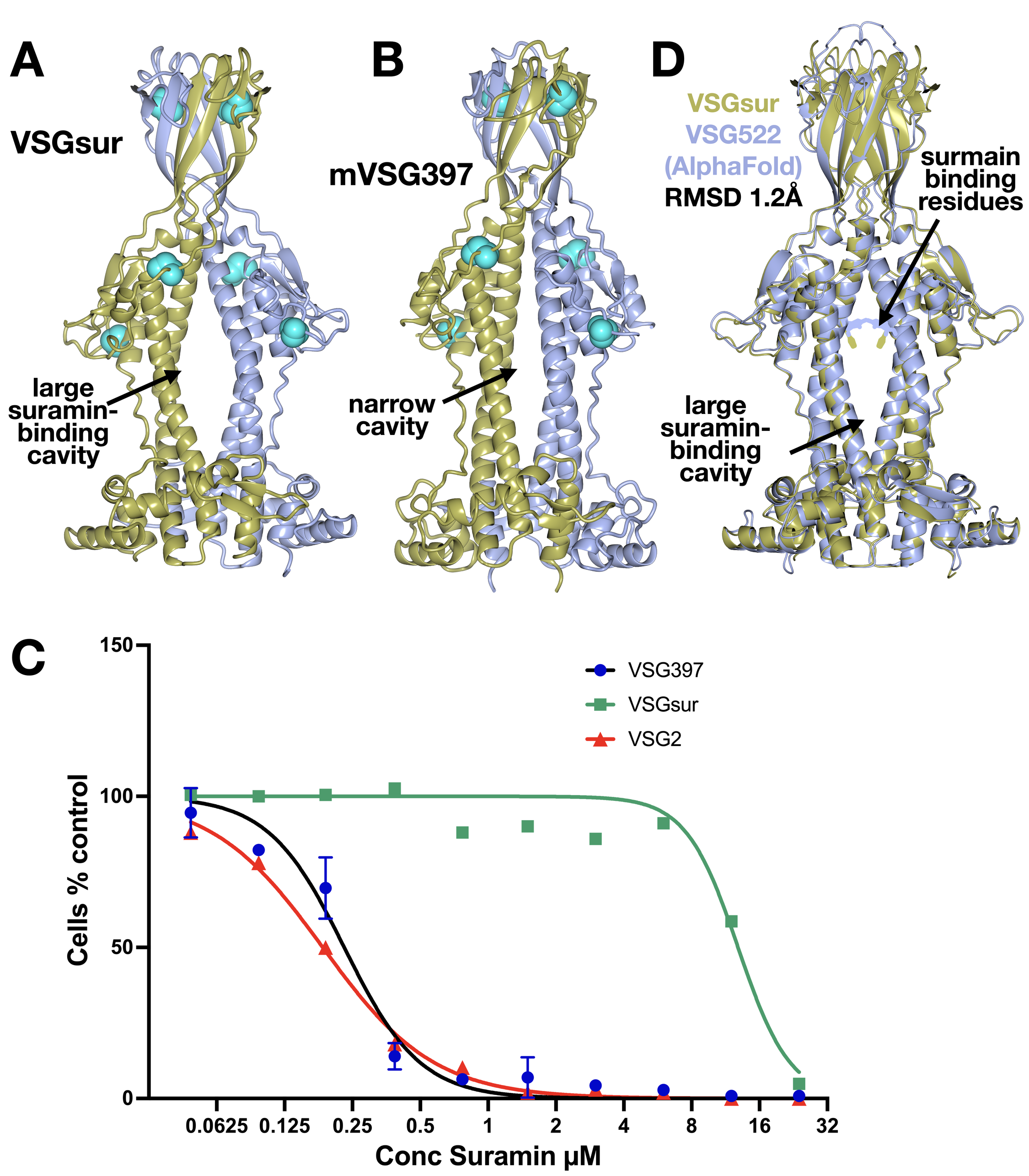

Supplement: S2 Fig — (A) and (B) Ribbon diagrams drawn with CCP4Mg showing that the pocket between monomers is much smaller in mVSG397 as compared to VSGsur. Note that the dimer of VSG397, like that of VSGsur shown, is crystallographic, as VSG397 crystallizes as a monomer in the asymmetric unit (like unbound VSGsur, PDB ID 6Z7A). However, a standard class A dimer is present in the crystals with the dimeric axis aligned with a crystallographic two-fold axis of symmetry (again, as seen with unbound VSGsur). Such an arrangement, monomer in the asymmetric unit, alignment of the dimer axis with a crystallographic two-fold axis of rotation, has also been seen with VSG2. (C) Suramin resistance assays showing that mVSG397 does not confer resistance to suramin. Differences between VSG397 and VSG2 are not statistically significant (two-tailed P = 0.3029) whereas the difference between VSG397 and VSGsur are statistically significant (two-tailed P<0.0001). (D) AlphaFold was used to predict the structure of a VSG522 dimer with ColabFold. The panel shows an alignment of VSGsur (gold) and VSG522 (blue) with the key suramin-binding residues of VSGsur (H122 from each monomer) and the corresponding H93 of VSG522 colored as the main chains. (TIF) [file pntd.0011093.s002.tif]

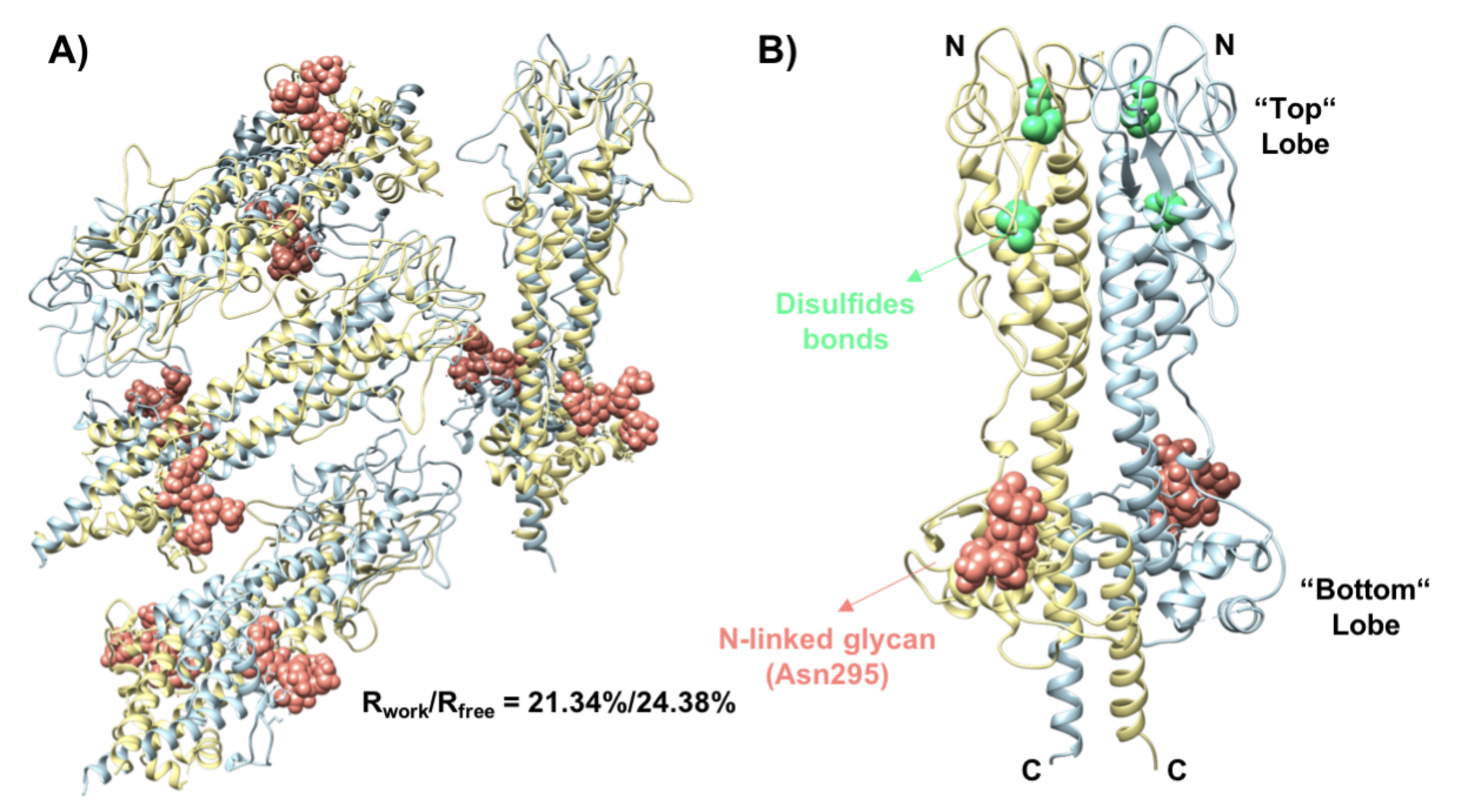

Supplement: S3 Fig — Molecular replacement was performed by PHASER-MR on the native crystal dataset using BSF VSG1 as search model to determine the structure of mVSG531 (Methods). (A) Eight molecules of mVSG531 was found in the crystal asymmetric unit. (B) N-linked glycan is attached at Asn295 at the dimer’s bottom lobe (salmon pink spheres). Two disulfide bonds are observed at the top lobe of each molecule (green spheres). Images of protein structures were generated and edited using CHIMERA. (TIF) [file pntd.0011093.s003.tif]

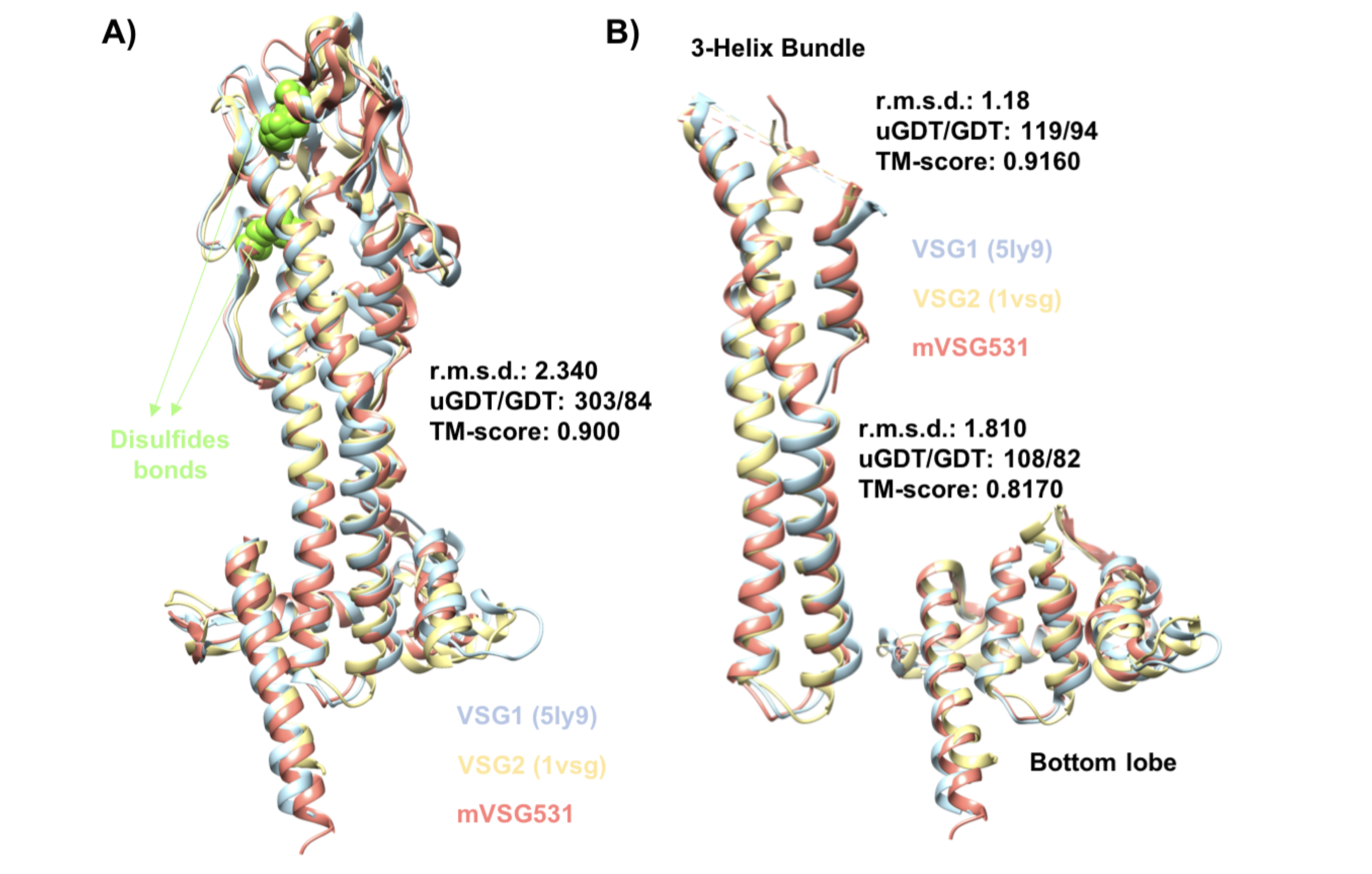

Supplement: S4 Fig — (A) Overall structural alignment of mVSG531, VSG2 (PDB code: 1VSG), and VSG1 (PDB code: 5LY9) monomers (yellow: VSG2, blue: VSG1, salmon pink: mVSG531, green: disulfide bonds) (B) Three-helix bundle core (left) and the bottom lobe (right) structural alignment. Structural alignment was performed by DeepAlign in RaptorX structure alignment server. (r.m.s.d.: root mean square deviation uGDT/GDT: (unnormalized) global distance test, TM-score: template modeling score). Images of protein structures were generated using CHIMERA. (TIF) [file pntd.0011093.s004.tif]

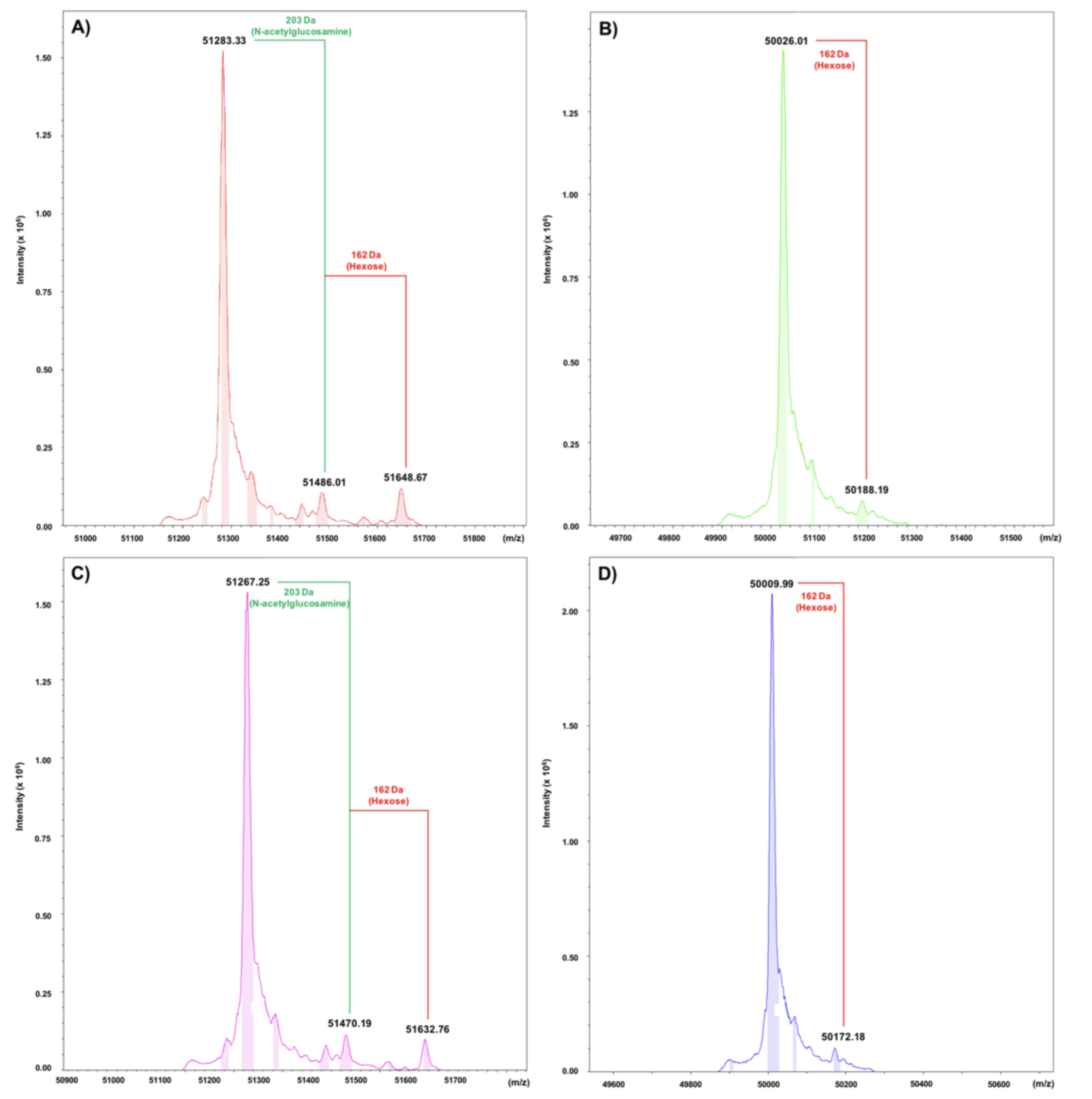

Supplement: S5 Fig — Intact mass spectrometry was performed on full length mVSG1954 wildtype and S321A (A and C). Both protein constructs are also treated with PNGaseF to cleave the N-linked glycans (B and D). Mass differences of 203 Da and 162 Da was observed in the full-length mVSG1954 wildtype (A), which correspond to N-acetylglucosamine and hexose molecules, respectively. When the sample was treated with PNGaseF (B), only the 162 Da mass difference was observed, indicating the loss of N-linked glycan and the possible presence of O-linked glycan. This was examined by mutating the putative O-glycosylation site at S321 to alanine, which cannot be O-glycosylated. A 16 Da mass difference was observed between the full length mVSG1954 wildtype (A) and S321A (C), indicating the serine to alanine mutation. However, when treated with PNGaseF (D), the 162 Da mass difference still presents, indicating that the protein is not O-glycosylated on that residue. Since the material is FL VSG1954, it is possible the loss of a mass equal to a hexose could be from the CTD GPI anchor or from another site on the NTD that was, nonetheless, not seen in the crystals. (TIF) [file pntd.0011093.s005.tif]

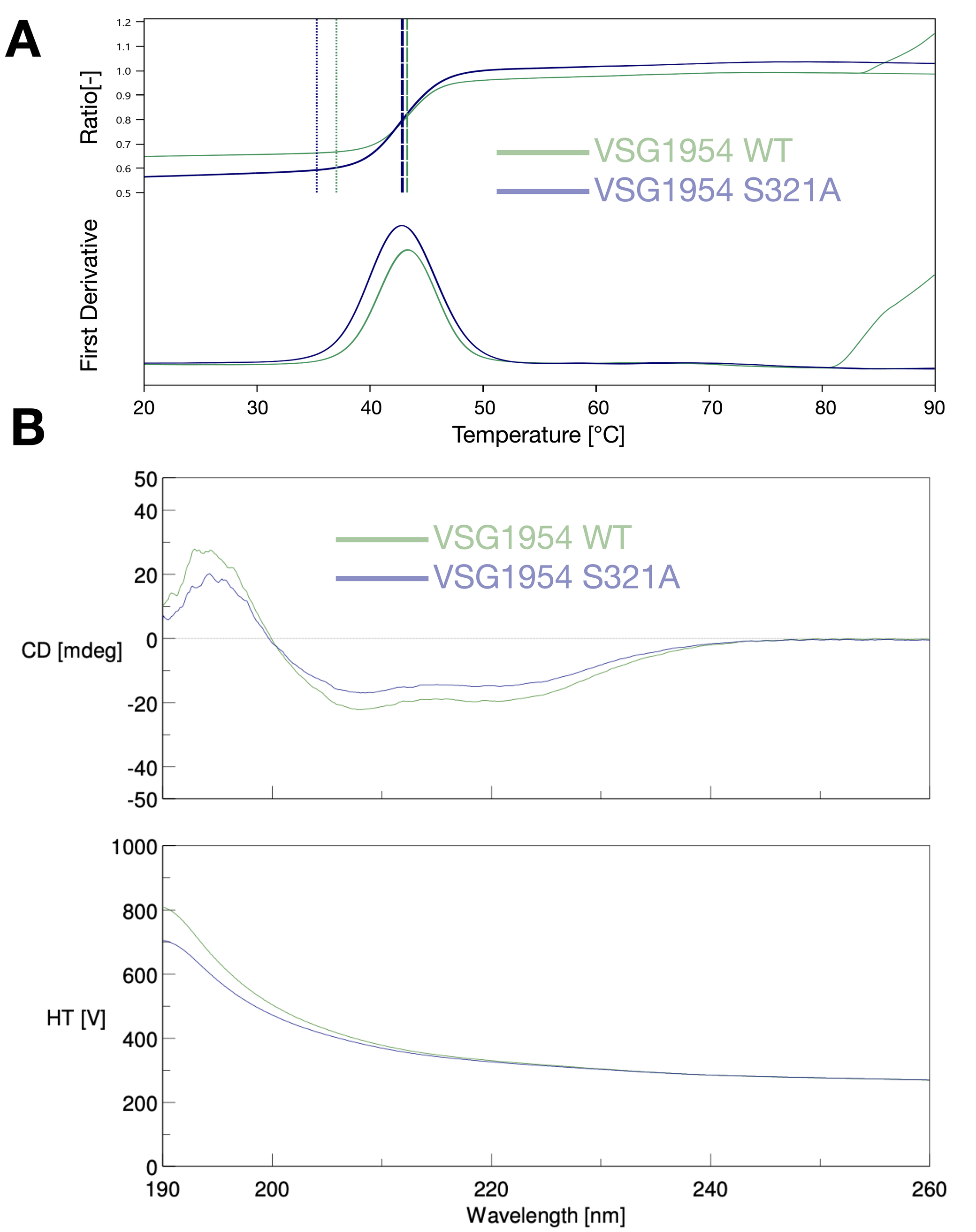

Supplement: S6 Fig — (A) thermal stability curves of the wild type (WT) mVSG1954 and the VSGS321A mutant (Methods). (B) Circular dichroism spectra of the WT and mutant VSG1954 (Methods). (TIF) [file pntd.0011093.s006.tif]
