# Supplementary material for: Structural similarities between the metacyclic and bloodstream form variant surface glycoproteins of the African trypanosome
Source: PLoS Negl Trop Dis. 2023 Feb 13;17(2):e0011093. doi: 10.1371/journal.pntd.0011093 (PMC9956791; doi:10.1371/journal.pntd.0011093)
Supplement: S1 Table — (DOCX) [file pntd.0011093.s007.docx]

**S1 Table: Crystallographic Statistics**

| **Parameter** | **VSG531** | **VSG1954** | **VSG397** |
| --- | --- | --- | --- |
| Wavelength | 1.00 Å | 0.9198 Å | 1.00 Å |
| Resolution range | 59.09-1.95  (2.021.95) | 60.29-1.68  (1.74-1.68) | 54.62-1.26  (1.305-1.26) |
| Space group | P 1 21 1 | P 3 2 1 | C 2 2 21 |
| Unit cell | 50.107 170.88 163.65  90 90.705 90 | 69.614 69.614 115.36  90 90 120 | 55.56 218.48 66.44  90 90 90 |
| Total reflections | 592350 (57625) | 723113 (71206) | 536522 (24839) |
| Unique reflections | 195472 (18665) | 37543 (3705) | 96793 (8421) |
| Multiplicity | 3.0 (3.1) | 19.3 (19.2) | 5.5 (2.9) |
| Completeness (%) | 97.68 (93.16) | 99.79 (97.89) | 96.37 (79.98) |
| Mean I/sigma(I) | 4.24 (1.13) | 7.45 (0.48) | 6.56 (0.42) |
| Wilson B-factor | 34.64 | 19.13 | 15.19 |
| R-merge | 0.1157 (0.7639) | 0.1453 (1.283) | 0.0879 (1.321) |
| R-meas | 0.1412 (0.9266) | 0.1492 (1.318) | 0.0972 (1.575) |
| R-pim | 0.07975 (0.5179) | 0.03383 (0.2986) | 0.04045( 0.8407) |
| CC1/2 | 0.977 (0.629) | 0.999 (0.772) | 0.99 (0.219) |
| CC* | 0.994 (0.879) | 1 (0.934) | 0.998 (0.599) |
| Reflections (refinement) | 195256 (18624) | 37464 (3627) | 95996 (7883) |
| Reflections (R-free) | 9605 (847) | 1877 (201) | 4807 (404) |
| R-work | 0.2134 (0.3583) | 0.1757 (0.3505) | 0.1834 (0.3799) |
| R-free | 0.2438 (0.3808) | 0.2137 (0.3762) | 0.2160 (0.4115) |
| CC(work) | 0.916 (0.772) | 0.968 (0.829) | 0.960 (0.596) |
| CC(free) | 0.904 (0.745) | 0.971 (0.783) | 0.973 (0.596) |
| Non-hydrogen atoms | 22693 | 2798 | 3366 |
| macromolecules | 20778 | 2514 | 2882 |
| ligands | 618 | 62 | 53 |
| solvent | 1339 | 250 | 456 |
| Protein residues | 2866 | 353 | 389 |
| RMS(bonds) | 0.006 | 0.011 | 0.009 |
| RMS(angles) | 1.16 | 1.34 | 1.21 |
| Ramachandran favored (%) | 96.78 | 98.84 | 98.44 |
| Ramachandran allowed (%) | 3.22 | 1.16 | 1.30 |
| Ramachandran outliers (%) | 0.00 | 0.00 | 0.26 |
| Rotamer outliers (%) | 0.25 | 0.41 | 0.68 |
| Clashscore | 1.81 | 3.19 | 1.55 |
| Average B-factor | 46.61 | 29.42 | 25.88 |
| macromolecules | 47.27 | 28.74 | 24.30 |
| ligands | 30.97 | 61.13 | 67.38 |
| solvent | 43.09 | 31.97 | 33.92 |
| Number of TLS groups | 45 | 5 | 5 |

Statistics for the highest-resolution shell are shown in parentheses.
